# Revising Incidence and Mortality of Lung Cancer in Central Europe: An Epidemiology Review From Hungary

**DOI:** 10.3389/fonc.2019.01051

**Published:** 2019-10-23

**Authors:** Krisztina Bogos, Zoltán Kiss, Gabriella Gálffy, Lilla Tamási, Gyula Ostoros, Veronika Müller, László Urbán, Nóra Bittner, Veronika Sárosi, Aladár Vastag, Zoltán Polányi, Zsófia Nagy-Erdei, Zoltán Vokó, Balázs Nagy, Krisztián Horváth, György Rokszin, Zsolt Abonyi-Tóth, Judit Moldvay

**Affiliations:** ^1^Department of Pulmonology, National Korányi Institute of Pulmonology, Budapest, Hungary; ^2^MSD Pharma Hungary Ltd., Budapest, Hungary; ^3^Pulmonology Hospital, Törökbálint, Hungary; ^4^Department of Pulmonology, Semmelweis University, Budapest, Hungary; ^5^Mátraháza Healthcare Center, University Teaching Hospital, Mátraháza, Hungary; ^6^Pulmonology Clinic, University of Debrecen, Debrecen, Hungary; ^7^Faculty of Medicine, University of Pécs, Pécs, Hungary; ^8^Department of Health Policy and Health Economics, Eötvös Loránd University, Budapest, Hungary; ^9^RxTarget Ltd., Szolnok, Hungary; ^10^University of Veterinary Medicine, Budapest, Hungary; ^11^Department of Tumor Biology, National Korányi Institute of Pulmonology, Semmelweis University, Budapest, Hungary; ^12^2nd Department of Pathology, MTA-SE NAP, Brain Metastasis Research Group, Hungarian Academy of Sciences, Semmelweis University, Budapest, Hungary

**Keywords:** lung cancer, incidence, mortality, epidemiology, European standard population, Hungary

## Abstract

**Objective:** While Hungary is often reported to have the highest incidence and mortality rates of lung cancer, until 2018 no nationwide epidemiology study was conducted to confirm these trends. The objective of this study was to estimate the occurrence of lung cancer in Hungary based on a retrospective review of the National Health Insurance Fund (NHIF) database.

**Methods:** Our retrospective, longitudinal study included patients aged ≥20 years who were diagnosed with lung cancer (ICD-10 C34) between 1 Jan 2011 and 31 Dec 2016. Age-standardized incidence and mortality rates were calculated using both the 1976 and 2013 European Standard Populations (ESP).

**Results:** Between 2011 and 2016, 6,996 – 7,158 new lung cancer cases were recorded in the NHIF database annually, and 6,045 – 6,465 all-cause deaths occurred per year. Age-adjusted incidence rates were 115.7–101.6/100,000 person-years among men (ESP 1976: 84.7–72.6), showing a mean annual change of − 2.26% (*p* = 0.008). Incidence rates among women increased from 48.3 to 50.3/100,000 person-years (ESP 1976: 36.9–38.0), corresponding to a mean annual change of 1.23% (*p* = 0.028). Age-standardized mortality rates varied between 103.8 and 97.2/100,000 person-years (ESP 1976: 72.8–69.7) in men and between 38.3 and 42.7/100,000 person-years (ESP 1976: 27.8–29.3) in women.

**Conclusion:** Age-standardized incidence and mortality rates of lung cancer in Hungary were found to be high compared to Western-European countries, but lower than those reported by previous publications. The incidence of lung cancer decreased in men, while there was an increase in incidence and mortality among female lung cancer patients.

## Introduction

Lung cancer was the most commonly diagnosed tumor in 2018 and the first cause of cancer death worldwide in both sexes (1, 2). Monitoring the incidence and mortality of lung cancer is essential for both the design and evaluation of public health interventions, as well as for the evaluation of treatment effectiveness.

Despite improving trends, Hungary is still reported to be among European countries with the highest incidence and mortality of lung cancer. An article series starting from 1990 published in the European Journal of Cancer (EJC) projected the incidence of lung cancer to be 109.3/100,000 standardized person-years (SPYs) among men and 46.5/100,000 SPYs among women in 2012 (2), and to be 111.6 and 58.7/100,000 SPYs, respectively in 2018 (3) using the ESP 1976 for standardization. Results from further epidemiology studies were in line with these observations regarding the position of Hungary in Europe (4). Youlden et al. even reported the highest worldwide incidence of lung cancer for Hungary, with a continuously increasing incidence among Hungarian women, but a decreasing trend in incidence among Hungarian men between 1995 and 2015 (5). Similarly to the high incidence of lung cancer in Hungary, mortality was also reported to be high in the EJC report series: mortality rates were 96.4/100,000 person-years in men and 37.7/100,000 person-years in women in 2012 (2). These publications used WHO GLOBOCAN as a source of Hungarian lung cancer mortality rates which receives regular inputs from the Hungarian Central Statistician Office (CSO). The CSO reports annual mortality rates for all cancer types in Hungary including lung cancer based on death certificates^1^. Between 2011 and 2016, 33,093 male and 19,296 female deaths were recorded in the database with lung cancer registered as the cause of death.

While Hungary is often reported to have the highest incidence and mortality rates of lung cancer, Hungary did not report any nationwide incidence rates during the last decades, and no exact clinical data source and explanation were provided to support the trends mentioned above. Therefore, the main objective of our study was to explore the incidence, prevalence and mortality rates of lung cancer in Hungary between 2011 and 2016 based on data source similar to Western European countries' national clinical registries, and to explore trends in both genders.

## Materials and Methods

### Study Design

Our retrospective, longitudinal study was performed using the database of the National Health Insurance Fund of Hungary (NHIF). The NHIF database is a nationwide insurance system covering close to 100% of the Hungarian population, which collects patient ID and ICD-10 code information about all in- and out-patient visits, as well as about all prescription of drugs which are reimbursed in Hungary. The study was approved by the National Ethical Board for Health Research (10338-5/2019/EKU).

Our analysis included patients with lung cancer (ICD-10:C34) who were newly diagnosed between 1 Jan. 2011 and 31 Dec. 2016 and were above 20 years of age at the time of diagnosis (Table 1). To avoid the potential miscoding of lung cancer, we included patients with a minimum of two occurrences of the ICD-10 code C34 within more than 30 but <365 days. This ensured that we only included patients who were actually involved in lung cancer care. One occurrence of C34 was also accepted if a patient died within 60 days after the first C34 code, when the repeated occurrence of the ICD code C34 was not applicable. To avoid the inclusion of patients with lung metastases from a different primary tumor due to miscoding, we excluded patients with cancer-related ICD-10 codes other than C34 and those receiving anticancer therapy other than lung cancer treatment within 6 months before or 12 months after the first occurrence of C34. The 3-years period between 2008 and 2010 was considered as a reference period to detect newly diagnosed lung cancer patients in 2011. Data on the size of the Hungarian population for incidence and prevalence calculations by age and sex were obtained from the Hungarian CSO. The dates and numbers of deaths among lung cancer patients were also collected from the NHIF database.

**Table 1:** Inclusion, exclusion criteria.

**Inclusion criteria**
Newly diagnosed lung cancer between 1 Jan. 2011 and 31 Dec. 2016
Two occurrences of the ICD-10 code C34 within more than 30 days but <365 days from diagnosis
One occurrence of the ICD-10 C code 34 if a patient died within 60 days after the first C34 code
Age above 20 years at diagnosis
**Exclusion criteria**
Cancer-related ICD-10 codes other than C34 within 6 months before or 12 months after diagnosis
Anticancer therapy other than lung cancer treatment protocols within 6 months before or 12 months after the first lung cancer ICD-10 code

The annual numbers of newly diagnosed lung cancer patients are presented as crude numbers (*n*), and as incidence rates per 100,000 persons at risk. The annual number of deceased patients was also determined. All-cause mortality was expressed as crude numbers of death and crude all-cause mortality rates per 100,000 person-years in the respective sex- and age-specific group of the population. We also calculated standardized incidence and mortality rates. Incidence and mortality data were adjusted for age using the ESP from 2013 (6) and from 1976 to allow for direct comparisons with earlier publications. Where crude numbers of any parameter were recorded below 10, we indicated “ <10” as the Hungarian NHIF data protection laws does not permit the presentation of case numbers below 10 in a stratum. In these cases, calculations were run on the exact crude numbers.

### Statistical Analysis

Linear regression was applied to estimate the annual change of the mean age of patients. The outcome was age in years, the explanatory variable was the year. Poisson regression was used to estimate the annual change of the crude incidence and mortality. The outcome was the number of patients, the offset was the log of the number of patients at risk (in the case of incidence) and the mid-year population published by Hungarian Central Statistical Office (in the case of prevalence), the explanatory variables were the year, age group, gender, and their paired interactions. The size of the at-risk population was determined based on the difference between mid-year population sizes and the number of previously diagnosed lung cancer patients on 1 January each year. The same method was used for incidence, prevalence, and mortality rates. The outcomes were the rates for 100,000 person-years standardized for the European population (standard ESP 2013).

In all regression models mentioned above we estimated annual trends with 95% confidence intervals (95% CI). As data were not independent (e.g., the same person was in the diseased population in different years), a block-based bootstrap method was used for time series with a fixed block size of two.

We also studied the association between smoking prevalence in the population and the incidence and mortality of lung cancer in an ecological analysis. For age-standardized incidence and mortality rates per 100,000 person-years, the source of regression analysis was the age-standardized incidence rate of lung cancer in European countries reported by Ferlay et al. and the prevalence of smoking prevalence in the population aged ≥15 years based on a WHO report from 1998 to 2002 (7). Significance of correlation was not calculated; Figure 4 was created to demonstrate the group of countries which are within and out of the 95% predictive range. All calculations were performed with R version 3.5.2 (20/12/2018) with package boot version 1.3-20.

## Results

### Crude Numbers

In 2011, 7,158 new lung cancer cases were registered in the NHIF database, while in 2016 we found 6,996 new cases resulting in a crude annual rate of 71.9 to 71.4 per 100,000 person-years during the 6-year study period. The proportion of male patients decreased from 63.17% to 59.69% from 2011 to 2016. The mean age at diagnosis was 64.5 years in men (*SD* ± 9.85) and 64.9 years in women (*SD* ± 11.2) in 2011, which increased to 65.8 years (*SD* ± 9.41) and 66.0 years (*SD* ± 10.5) (Table 2 and Supplementary Table 1).

**Table 2:** Crude incidence, and mortality of lung cancer in Hungary between 2011 and 2016.

	**Number of patients**												**Mean annual change % (95%CI)**	***p*-value**
	**2011**		**2012**		**2013**		**2014**		**2015**		**2016**			
Patients with new LC diagnosis (*n*)	7,158		6,924		6,856		6,949		6,981		6,996			
Male (*n*, % of LC patients)	4,522	63.17%	4,307	62.20%	4,126	60.18%	4,227	60.83%	4,138	59.28%	4,176	59.69%	−3.02 (−5.77 to −1.14)	0.0193
Mean age at diagnosis (y, mean ±*SD*)	64.66	±10.37	65.09	±10.39	65.15	±10.41	65.41	±10.17	65.64	±9.90	65.88	±9.84	0.23 (0.17–0.24)	<0.0001
Male (y, mean ±*SD*)	64.51	±9.85	64.85	±9.83	64.89	±9.95	65.32	±9.68	65.58	±9.48	65.80	±9.41	0.26 (0.20–0.33)	<0.0001
Female (y, mean ±*SD*)	64.93	±11.19	65.49	±11.23	65.56	±11.06	65.54	±10.89	65.72	±10.48	65.99	±10.45	0.17 (0.03–0.27)	0.0046
LC Patients died (*n*, crude rate per 100.000)	6,045		6,208		6,154		6,283		6,273		6,465			
Male (*n*, % of LC patients)	3,946	65.28%	4,059	65.38%	3,956	64.28%	3,988	63.47%	3,861	61.55%	4,088	63.23%	−2.79 (−5.34 to −1.62)	0.0456
Mean age at death (y, mean ±*SD*)	66.46	±10.35	66.67	±10.32	66.89	±10.20	67.04	±10.16	67.34	±9.92	67.95	±9.68	0.27 (0.19–0.43)	<0.0001
Male (y, mean ±*SD*)	66.09	±9.83	66.30	±9.97	66.54	±9.66	66.66	±9.79	66.97	±9.49	67.55	±9.37	0.27 (0.19–0.42)	<0.0001
Female (y, mean ±*SD*)	67.16	±11.22	67.38	±10.93	67.51	±11.10	67.70	±10.73	67.92	±10.54	68.64	±10.14	0.26 (0.17–0.43)	<0.0001

*CI, confidence interval; LC, lung cancer; SD, standard deviation*.

The annual number of patients who died of lung cancer was between 6,045 (2011) and 6,465 (2016) during the study period (Table 2 and Supplementary Table 2). The mean age at the time of death increased from 66.5 (*SD* ± 10.4) to 68.0 (*SD* ± 9.68) years between 2011 and 2016, and was higher among females during the whole study period (67.2–68.6 years vs. 66.1–67.6 years in males).

### Incidence Rates

**Figure 1:**
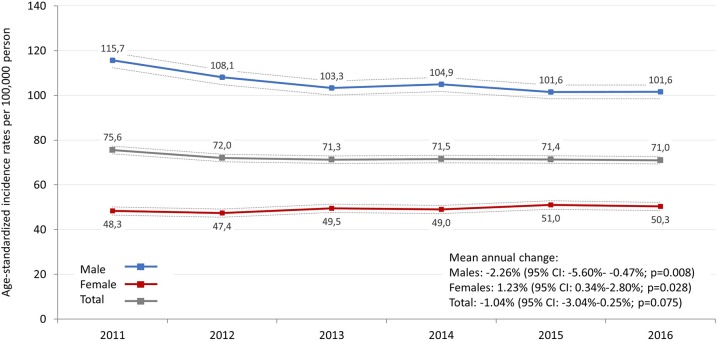
Age-standardized incidence rates (standard: ESP 2013) of lung cancer by sex in Hungary between 2011 and 2016 (per 100,000 person-years; dotted lines represent 95% CI). CI, confidence interval.

A continuous and significant decrease was detected in the incidence rate of lung cancer in men between 2011 and 2016. In 2011, the age-adjusted incident rate (standard: ESP 2013) was 115.7/100,000 person-years (95% CI: 112.3–119.1), while in 2016 it was 101.6/100.000 person-years (95% CI: 98.5–104.7), resulting in an absolute change of −13,9% (Figure 1, Supplementary Table 3). On the other hand, incidence rates in women increased from 48.3 to 50.3 per 100,000 person-years (95% CI: 46.5–50.2 and 48.5–52.2), showing a mean annual change of 1.23% (*p* = 0.028). Consequently, we did not find any significant change in incidence rates in the whole lung cancer population during the study period (*p* = 0.075). When applying ESP 1976, age-standardized incidence rates were 84.7 (in 2011) and 72.6 (in 2016) per 100,000 person-years in male lung cancer patients and 36.9 (in 2011) and 38.0 (in 2016) in female patients (Supplementary Table 3).

### Mortality Rates

**Figure 2:**
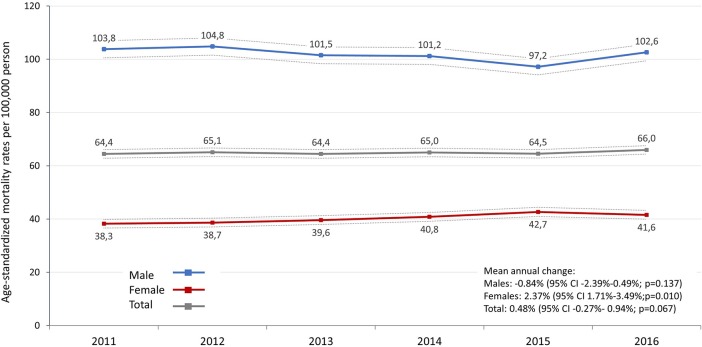
Age-standardized mortality rates (standard: ESP 2013) of lung cancer by sex in Hungary between 2011 and 2016 (per 100,000 person-years; dotted lines represent the 95% CI). CI, confidence interval.

The age-standardized mortality rate (standard: ESP 2013) of lung cancer varied between 103.8 (95% CI: 100.52–107.00) and 97.2 (95% CI: 94.12–100.25) per 100,000 person-years in men and between 38.3 (95% CI: 36.6–39.9) and 42.7 (95% CI: 41.0–44.4) in women (Figure 2). There was no significant change in age-standardized mortality rates in male patients from 2011 to 2016, in contrast, age-standardized mortality rates significantly increased by 2.4% in women (95% CI: 1.7%–3.5%; *p* = 0.01) (Supplementary Table 3). We did not find any significant changes in age-standardized mortality rates in the total patient population (mean annual change: 0.48%; 95% CI: −0.27%–0.94%; *p* = 0.067). When using ESP 1976, age-standardized mortality rates were found to be 72.8–69.7 among males and 27.0–29.3 among females (Supplementary Table 3).

**Figure 3:**
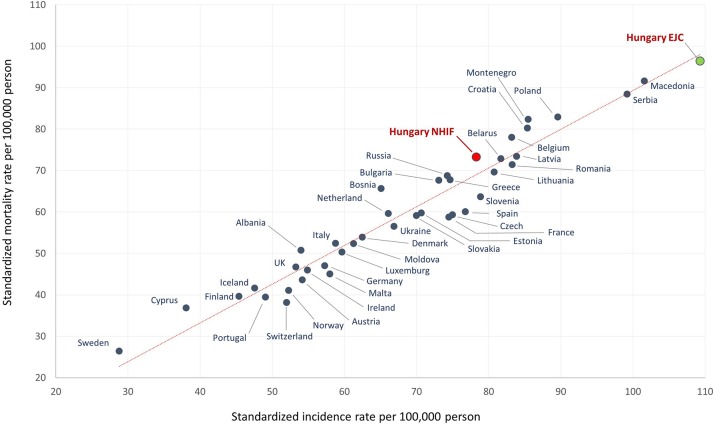
Age-standardized incidence and mortality rates per 100,000 person-years among male lung cancer patients in European countries and Hungary based on the NHIF survey in 2012 (using ESP 1976). Description: A dot in the graph shows age-standardized incidence and mortality rates together for a country. The Y axis represents the standardized mortality rate per 100,000 population, while the X axis represents the standardized incidence rate per 100,000 population.

Using ESP 1976 for standardization calculations to compare our results to previously reported values for Hungary, the age-standardized incidence rate per 100,000 person-years in men was 78.3 and age-standardized mortality was 72.8 in 2012. These results are different from those reported by Ferlay et al. for European countries from the same year, 2012 (109.3 and 96.4), as seen in Figure 3 and Supplementary Table 3. In Figure 3, a dot shows age-standardized incidence and mortality rates together for a country. The Y axis represents the standardized mortality rate per 100,000 population, while the X axis represents the standardized incidence rate per 100,000 population. Among women, there was also a considerable difference between our results based on the NHIF database and the publication by Ferlay et al.: we found an incidence rate of 35.7 and a mortality rate of 28.0 in 2012 (Supplementary Table 3), while those reported by Ferlay et al. were 46.5 and 37.7, respectively (Supplementary Figure 1).

**Figure 4:**
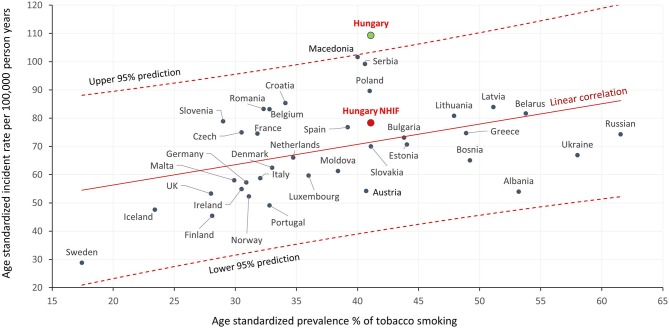
Correlation between the age-standardized incidence rate of lung cancer in European countries in 2012 based on the report by Ferlay et al. (2) and the age-standardized prevalence (%) of tobacco smoking in the population aged ≥15 years in 1998–2002. Description: incidence rates of lung cancer are presented as a function of the prevalence of tobacco smoking. The X axis represents the age-standardized prevalence of smoking expressed in percentages in the population aged >15 years between 1998 and 2002 and the Y axis represents the age-standardized incidence rates of different European countries, thus merging these data in one dot.

In the ecological study of age-standardized incidence rates of European countries in 2012 from Ferlay in relation to smoking prevalence in 1998–2002 based on WHO data, only the Hungarian incidence rate was out of the 95% predictive range as seen in Figure 4. On the other hand, using our results based on the NHIF database, the Hungarian value fell within the range. (In Figure 4, incidence rates of lung cancer are presented as a function of the prevalence of tobacco smoking. The X axis represents the age-standardized prevalence of smoking expressed in percentages in the population aged >15 years between 1998 and 2002 and the Y axis represents the age-standardized incidence rates of different European countries, thus merging these data in one dot).

## Discussion

Our nationwide, retrospective, longitudinal study provides comprehensive information on the epidemiology of lung cancer in Hungary using the database of the NHIF.

The main findings of this large-scale evaluation can be summarized as follows:

1. Hungarian age-standardized incidence and mortality rates of lung cancer were high but lower than those reported in earlier publications both among men and women.
2. The age-standardized incidence of lung cancer significantly decreased in men, while we found a significant increase among women during the study period.

The decreasing trend in the incidence of lung cancer in men is well-documented, which was preceded by a decreasing trend in smoking starting from the 1950s (8, 9). We found similar trends in our nationwide NHIF database study where incidence rates among men significantly decreased from 115.7 to 101.6 per 100,000 person-years between 2011 and 2016, corresponding to a decrease of 2.35%.

Differences in trends among males vs. females could be explained by a number of factors including genetic and epigenetic differences, gender-specific lifestyle behaviors, or differences in sex hormone activity (10). However, it can be concluded that the most important risk factor for the development of lung cancer is smoking in both genders (11, 12). Smoking among women has significantly increased since the 1960s, resulting in a consequent increase in the risk of lung cancer in the female population (13). According to a Hungarian survey on smoking prevalence published in 2010, 40.6% of males and 31.7% of females smoked regularly or occasionally (14). When comparing their results to previous local reports, the authors of this publication found a stagnant trend in smoking among men and an increasing trend among women. A new anti-smoking law was launched in Hungary in 2011, and the Methodological Support Center for Smoking Cessation opened its gates at the National Korányi Institute of Pulmonology in 2012 to monitor the efficacy of the new regulation. In their latest report in 2013, they confirmed previous findings on smoking habits in Hungary: the proportion of male smokers showed a significant decrease (to 32%), while the prevalence of smoking among women was stable during the observation period (24–25%) (15). This changing prevalence of smoking is also in line with our findings showing a decreasing difference in the risk of lung cancer incidence and mortality between the two sexes in all age cohorts.

The mean age at the diagnosis of lung cancer was 64.7 years in 2011, which increased to 65.9 by the end of the 6-years study period. The mean age of patients at the time of death was about 2 years higher than the mean age at diagnosis, and showed an increasing trend between 2011 and 2016 (from 66.5 to 68.0 years). Our study supports previous findings showing an increase in the mean age at the time of lung cancer diagnosis (16).

One of the main findings of our nationwide study is that the incidence and mortality rates of lung cancer among men and women were definitely lower than those reported in previous publications. The most commonly cited source which reported the highest incidence of lung cancer in Hungary is the article series by Ferlay et al. (3). These reports used statistical models to estimate incidence and mortality rates at 25 cancer sites in 40 European countries based on published national cancer registry data where available, and estimated rates wherever no exact data were found. Hungary did not report any nationwide incidence rates during the study period of Ferlay et al., hence the rates were calculated based on WHO mortality data by approximating with neighboring countries' incidence to mortality rate ratio. Furthermore, while several countries collected incidence and mortality data from cancer or lung cancer registries, only mortality data were available from Hungary based on post-mortem reports of the CSO. It is important to note that cancer diagnosis rates are reported to be higher in autopsy findings compared to clinical findings, therefore autopsy rates for hospital deaths influence the probability of identifying lung cancer. A recent study in the Czech Republic, another Central European country, found a 44% higher incidence of lung cancer in autopsy reports compared to clinical settings (17). Previous publications also reported a 11–28% higher incidence of cancer in autopsy reports than those reported by clinicians (18, 19). Based on a WHO report, Hungary is one of the countries with the highest autopsy rates for hospital deaths: dissection is performed in 36.9% of patients who die at hospitals (20). On the other hand, some of the countries with the lowest reported incidence and mortality of lung cancer only have autopsy rates of 3.2% (Italy), 11.1% (Norway), and 8.4% (Sweden) (20). Consequently, the methodology applied by Ferlay may have played an important part in the overestimation of incidence rates in Hungary.

The article series published in EJC projected lung cancer incidence and mortality rates to be 109.3 and 96.4 among men and 46.5 and 37.7 per 100,000 person-years among women in 2012 using ESP 1976 (2). Incidence and mortality rates in our study were 78.3 and 72.8 among men and 35.7 and 28.02 among women, respectively (with ESP 1976), corresponding to 28.4–24.5% and 23.2–25.7% higher differences, which could be relevant when considering the position of Hungary regarding lung cancer rates among European countries.

In the European Health Information Gateway report (WHO Europe), the age-standardized prevalence of smoking among Hungarian men was found to be lower in the population aged ≥15 years than in several other Eastern European as well as Balkan countries including Serbia, Romania and Montenegro (7). Similar differences were seen regarding smoking prevalence among women: Hungary was not among the highest ranked countries. Although several other risk factors were identified for lung cancer (9, 10), smoking is still one of the main risk factors. Considering this, incidence and mortality rates of lung cancer may be similar or even lower in Hungary than in these countries, and not the highest in Europe. This hypothesis is supported by our analysis of correlation between the incidence of lung cancer and the prevalence of smoking (as seen in Figure 4): Hungarian data reported by Ferlay were outside the 95% predictive range, while our results based on the NHIF database were closer to the correlation trend line, within the 95% predictive range.

To summarize, relevant differences can be observed between the present findings and previous reports, which could be explained by differences in data collection methodology and the high autopsy rate for hospital deaths in Hungary (20) leading to a higher incidence of lung cancer diagnosis compared to countries where incidence data are derived from clinical registries (1–3).

Our study has certain strengths and limitations. The robust number of identified lung cancer patients, the carefully cleaned data, the 6-year-long follow-up period, the nationwide nature of the NHIF database all provide a solid foundation for drawing conclusions from our analysis. Nevertheless, the applied exclusion criteria may have led to the exclusion of patients who had other cancer types besides lung cancer. However, according to our estimations, the size of this patient population was not significant (21). Moreover, the NHIF database does not contain data on the histological type of lung cancer, on TNM stage, or the ECOG status of patients, and no laboratory results were available. Recognizing that mortality and prevalence rates vary significantly according to TNM stage and histological type, we did not try to compare our results with findings where data were stratified according to these parameters. Instead, we made comparisons with reports that provided overall lung cancer epidemiology data. In our study, we aimed to highlight the importance of differences in data collection methodology, therefore we had to use overall lung cancer incidence and mortality rates.

In conclusion, our study is the first nationwide investigation which describes the incidence and mortality rates of lung cancer in Hungary, showing lower rates than those reported in previous reports which were typically based on estimations. It should be emphasized that although the incidence and mortality of lung cancer are probably lower in Hungary than previously reported, they are still very high. Our analysis also highlights the importance of using datasets of similar quality and similar methodology of estimation for international comparisons. Our study could also provide guidance on the revision and/or validation of previous reports on lung cancer in countries where lung cancer registries were not available.

## Data Availability Statement

All datasets generated for this study are included in the manuscript/Supplementary Files.

## Ethics Statement

The studies involving human participants were reviewed and approved by National Ethical Board for Health Research, Hungary (10338-5/2019/EKU). Written informed consent for participation was not required for this study in accordance with the national legislation and the institutional requirements.

## Author Contributions

All authors listed have made a substantial, direct and intellectual contribution to the work, and approved it for publication.

### Conflict of Interest

ZK, AV, ZN-E, and ZP are employees of MSD Pharma Hungary Ltd. KH is a research fellow at Eötvös Loránd University, in employed in the framework of a joint research programme of Eötvös Loránd University and MSD Pharma Hungary Ltd. BN and ZV are employees of Eötvös Loránd University where their contribution to this project was financially compensated. KB, JM, and GO are employees of National Korányi Institute of Pulmonology. GG is employee of Oncology Center of Törökbálint. LT and VM are employees of Semmelweis University. LU is employee of Mátra Gyógyintézet. NB is employee of University of Debrecen. VS is employee of University of Pécs, where their contribution to this project was not financially compensated. GR and ZA-T are employees of RxTarget Ltd., where their contribution to this project was financially. The programme is financed by MSD Pharma Hungary Ltd.

## Footnotes

^1^http://statinfo.ksh.hu/Statinfo/haDetails.jsp (accessed June 6, 2019).
