# Supplementary material for: Revising Incidence and Mortality of Lung Cancer in Central Europe: An Epidemiology Review From Hungary
Source: Front Oncol. 2019 Oct 23;9:1051. doi: 10.3389/fonc.2019.01051 (PMC6819432; doi:10.3389/fonc.2019.01051)
Supplement: Supplementary file 1 [file Data_Sheet_1.docx]

**SUPPLEMENTARY FIGURES AND TABLES**


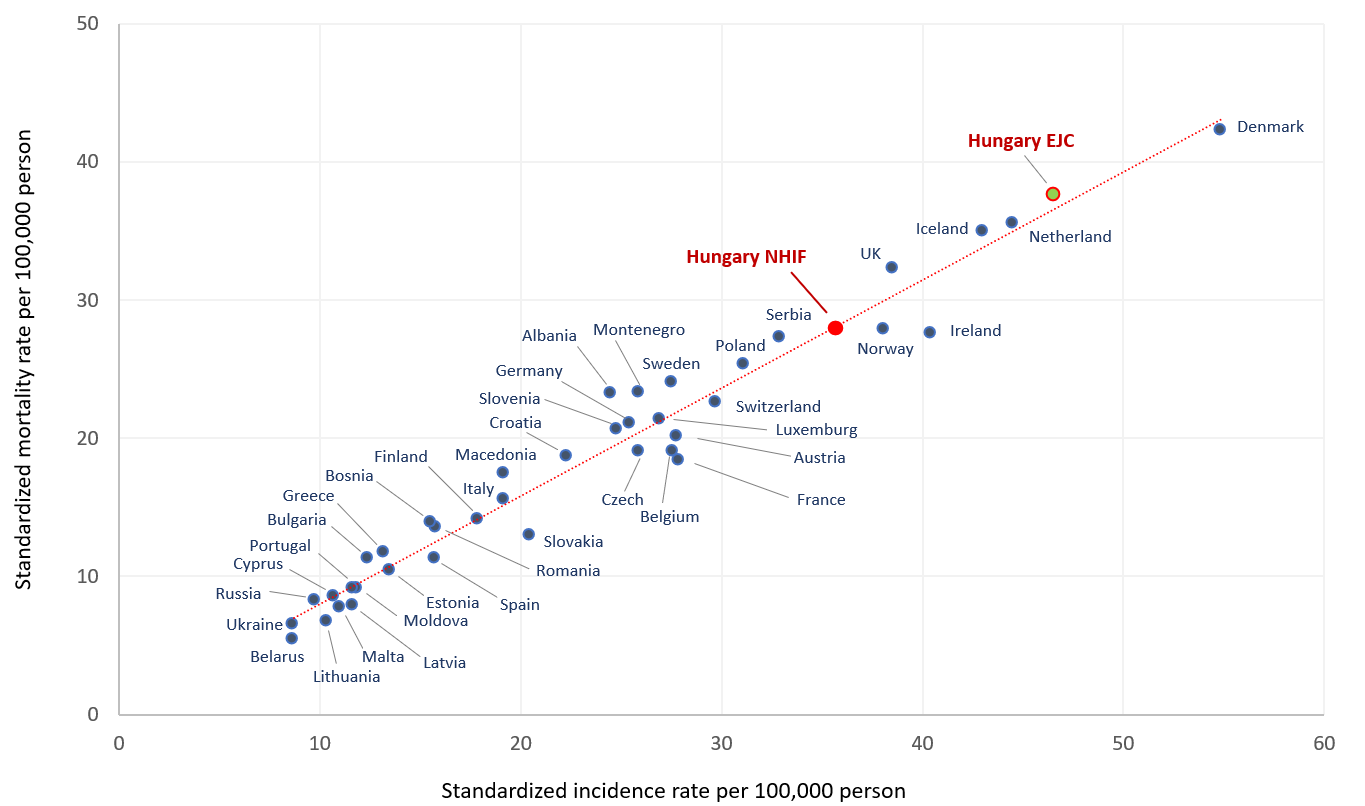


**Supplementary Figure 1** Age-standardized incidence and mortality rates per 100,000 person-years among female lung cancer patients in European countries and Hungary based on the NHIF survey in 2012 (using ESP 1976). Description: A dot in the graph shows age-standardized incidence and mortality rates together for a country. The Y axis represents the standardized mortality rate per 100,000 population, while the X axis represents the standardized incidence rate per 100,000 population.

**Supplementary Table 1** Number of patients with new diagnosis of lung cancer in Hungary by age and sex between 2011 and 2016

|  | **Number of Patients** | | | | | |
| --- | --- | --- | --- | --- | --- | --- |
| **Age group Total LC patients (n)** | **2011** | **2012** | **2013** | **2014** | **2015** | **2016** |
| 20-39 | 52 | 49 | 55 | 50 | 46 | 39 |
| 40-49 | 396 | 371 | 360 | 342 | 282 | 299 |
| 50-59 | 2,051 | 1,871 | 1,822 | 1,737 | 1,635 | 1,542 |
| 60-69 | 2,513 | 2,499 | 2,437 | 2,659 | 2,803 | 2,867 |
| 70-79 | 1,569 | 1,496 | 1,579 | 1,548 | 1,629 | 1,651 |
| 80-89 | 528 | 598 | 546 | 556 | 520 | 536 |
| 90≤ | 49 | 40 | 57 | 57 | 66 | 62 |
| **Age group Males (n)** | **2011** | **2012** | **2013** | **2014** | **2015** | **2016** |
| 20-39 | 30 | 18 | 36 | 24 | 27 | 17 |
| 40-49 | 216 | 212 | 203 | 192 | 153 | 170 |
| 50-59 | 1,332 | 1,199 | 1,076 | 1,028 | 944 | 898 |
| 60-69 | 1,627 | 1,627 | 1,563 | 1,704 | 1,728 | 1,786 |
| 70-79 | 1,029 | 929 | 955 | 976 | 985 | 988 |
| 80-89 | 269 | 306 | 270 | 272 | 276 | 291 |
| 90≤ | 19 | 16 | 23 | 31 | 25 | 26 |
| **Age group Females (n)** | **2011** | **2012** | **2013** | **2014** | **2015** | **2016** |
| 20-39 | 22 | 31 | 19 | 26 | 19 | 22 |
| 40-49 | 180 | 159 | 157 | 150 | 129 | 129 |
| 50-59 | 719 | 672 | 746 | 709 | 691 | 644 |
| 60-69 | 886 | 872 | 874 | 955 | 1,075 | 1,081 |
| 70-79 | 540 | 567 | 624 | 572 | 644 | 663 |
| 80-89 | 259 | 292 | 276 | 284 | 244 | 245 |
| 90≤ | 30 | 24 | 34 | 26 | 41 | 36 |

**Supplementary Table 2** Number of patients diagnosed with lung cancer and died in Hungary between 2011 and 2016 by age and sex

|  | **Number of Patients Died** | | | | | |
| --- | --- | --- | --- | --- | --- | --- |
| **Age group Total LC patients (n)** | **2011** | **2012** | **2013** | **2014** | **2015** | **2016** |
| 20-39 | 13 | 18 | 14 | 22 | 18 | <10 |
| 40-49 | 243 | 241 | 229 | 227 | 197 | 160 |
| 50-59 | 1,521 | 1,504 | 1,427 | 1,351 | 1,231 | 1,132 |
| 60-69 | 2,047 | 2,169 | 2,194 | 2,351 | 2,396 | 2,554 |
| 70-79 | 1,558 | 1,553 | 1,585 | 1,601 | 1,713 | 1,858 |
| 80-89 | 609 | 675 | 632 | 668 | 639 | 670 |
| 90≤ | 54 | 48 | 73 | 63 | 79 | 82 |
| **Age group Males (n)** | **2011** | **2012** | **2013** | **2014** | **2015** | **2016** |
| 20-39 | <10 | 13 | 10 | 14 | 11 | <10 |
| 40-49 | 134 | 152 | 130 | 135 | 123 | 104 |
| 50-59 | 1,023 | 997 | 915 | 860 | 751 | 742 |
| 60-69 | 1,411 | 1,457 | 1,500 | 1,559 | 1,535 | 1,653 |
| 70-79 | 1,019 | 1,046 | 1,037 | 1,038 | 1,082 | 1,174 |
| 80-89 | 329 | 377 | 336 | 348 | 325 | 373 |
| 90≤ | 23 | 17 | 28 | 34 | 34 | 38 |
| **Age group Females (n)** | **2011** | **2012** | **2013** | **2014** | **2015** | **2016** |
| 20-39 | <10 | <10 | <10 | <10 | <10 | <10 |
| 40-49 | 109 | 89 | 99 | 92 | 74 | 56 |
| 50-59 | 498 | 507 | 512 | 491 | 480 | 390 |
| 60-69 | 636 | 712 | 694 | 792 | 861 | 901 |
| 70-79 | 539 | 507 | 548 | 563 | 631 | 684 |
| 80-89 | 280 | 298 | 296 | 320 | 314 | 297 |
| 90≤ | 31 | 31 | 45 | 29 | 45 | 44 |

**Supplementary Table 3** Age-standardized incidence and mortality rates of lung cancer in Hungary by sex and age-specific incidence rates between 2011 and 2016 using ESP 2013 and 1976 for standardization

| **Incidence Rate per 100,000 Person years (ESP 2013)** | | | | | | | | |
| --- | --- | --- | --- | --- | --- | --- | --- | --- |
| **Characteristics** | **2011** | **2012** | **2013** | **2014** | **2015** | **2016** | **Mean annual change %  (95% CI) p-value** | |
|  |  |  |  |  |  |  |  |  |
| Overall | 75.6 | 72.0 | 71.3 | 71.5 | 71.4 | 71.0 | -1.04 (-3.04-0.25) | 0.075 |
| Sex |  |  |  |  |  |  |  |  |
| Female | 48.3 | 47.4 | 49.5 | 49.0 | 51.0 | 50.3 | 1.23 (0.34-2.80) | 0.028 |
| Male | 115.7 | 108.1 | 103.3 | 104.9 | 101.6 | 101.6 | -2.26 (-5.60- -0.47) | 0.008 |
| **Incidence Rate per 100,000 Person years (ESP 1976)** | | | | | | | | |
| **Characteristics** | **2011** | **2012** | **2013** | **2014** | **2015** | **2016** | **Mean annual change %  (95% CI) p-value** | |
|  |  |  |  |  |  |  |  |  |
| Overall | 56.9 | 53.6 | 53.0 | 53.2 | 52.9 | 52.4 | -1.45(-3.3-0) | 0.027 |
| Sex |  |  |  |  |  |  |  |  |
| Female | 36.9 | 35.7 | 37.4 | 37.2 | 38.6 | 38.0 | 1.08 (-0.01-2.59) | 0.059 |
| Male | 84.7 | 78.3 | 74.6 | 75.5 | 72.8 | 72.6 | -2.68 (-5.75- -0.86) | 0.010 |
| **Mortality Rate per 100,000 Person years (ESP 2013)** | | | | | | | | |
| **Characteristics** | **2011** | **2012** | **2013** | **2014** | **2015** | **2016** | **Mean annual change %  (95% CI) p-value** | |
|  |  |  |  |  |  |  |  |  |
| Overall | 64.4 | 65.1 | 64.4 | 65.0 | 64.5 | 66.0 | 0.48 (-0.27-0.94) | 0.067 |
| Sex |  |  |  |  |  |  |  |  |
| Female | 38.4 | 38.8 | 39.8 | 41.0 | 42.9 | 41.8 | 2.37 (1.71-3.49) | 0.010 |
| Male | 103.8 | 104.8 | 101.5 | 101.2 | 97.2 | 102.6 | -0.84 (-2.39-0.49) | 0.137 |
| **Mortality Rate per 100,000 Person years (ESP 1976)** | | | | | | | | |
| **Characteristics** | **2011** | **2012** | **2013** | **2014** | **2015** | **2016** | **Mean annual change %  (95% CI) p-value** | |
|  |  |  |  |  |  |  |  |  |
| Overall | 46.4 | 46.7 | 46.0 | 46.5 | 45.7 | 46.1 | -0.19 (-0.89-0.00) | 0.369 |
| Sex |  |  |  |  |  |  |  |  |
| Female | 27.7 | 28.0 | 28.6 | 29.5 | 30.6 | 29.3 | 1.51 (-0.77-3.50) | 0.107 |
| Male | 72.8 | 72.8 | 70.5 | 70.2 | 66.8 | 69.7 | -1.35 (-2.58-0.00) | 0.047 |
